# The first genetically authenticated case of Leber hereditary optic neuropathy in Sri Lanka: a case report and review of the literature

**DOI:** 10.1186/s13256-023-03763-x

**Published:** 2023-02-04

**Authors:** Kawmadi Gunawardena, Vajira H. W. Dissanayake, Thashi Chang

**Affiliations:** 1 Professorial Unit in Medicine, National Hospital of Sri Lanka and Postgraduate Institute of Medicine, Colombo, Sri Lanka; 2 Department of Anatomy, Genetics and Bioinformatics, Faculty of Medicine, University of Colombo, Colombo, Sri Lanka; 3 Department of Clinical Medicine, Faculty of Medicine, University of Colombo, Colombo, Sri Lanka

**Keywords:** Mitochondrial, Variable penetrance, Male predominance, Blindness

## Abstract

**Introduction:**

Leber hereditary optic neuropathy is a genetic disease of mitochondrial inheritance characterized by bilateral irreversible vision loss, predominantly affecting males. We report the first genetically authenticated Sri Lankan case of Leber hereditary optic neuropathy, illustrating its characteristic features of male predominance and variable penetrance.

**Case presentation:**

A 15-year-old previously healthy Sri Lankan boy presented with painless progressive vision loss in his right eye, followed by vision loss in his left eye within 3 months. There was no history of drug or toxin exposure, or a family history of vision loss. His parents were nonconsanguineous. On examination, he could only perceive light. Funduscopy revealed bilateral optic atrophy. Routine hematological and biochemical blood tests, including inflammatory markers, were normal. Cranial magnetic resonance imaging was unremarkable. Optical coherence tomography, and the clinical presentation, suggested a diagnosis of Leber hereditary optic neuropathy, which was confirmed by detection of m.14484T > C pathogenic variant in the *MT-ND6* gene through targeted genetic analysis for the three common pathogenic variants in mitochondrial deoxyribonucleic acid. He was homoplasmic for the variant, and his asymptomatic mother and two female siblings were also found to be harboring the variant with homoplasmy.

**Conclusions:**

This case report is intended to increase awareness of Leber hereditary optic neuropathy, and highlights the need to consider this rare diagnosis in the appropriate clinical context. It also illustrates the phenomena of incomplete penetrance and male predominance, and suggests the possibility of an X-linked gene governing Leber hereditary optic neuropathy disease expression, which warrants further investigation.

## Introduction

Mitochondrial diseases are a cause of chronic human disease, with an estimated prevalence of 1 in 10,000 and a further 1 in 200 individuals being at-risk pathogenic variant carriers [1]. Leber hereditary optic neuropathy (LHON) is by far the most common mitochondrial disease with an estimated prevalence of ∼ 1 in 25,000 in the northeast of England [2] and 1 in 68,403 in Australia [3].

It was first described in 1871 by the German ophthalmologist Theodore Leber (1840−1917) as a distinct clinical entity [4]. Limited data on prevalence suggest variability among different populations [5]. However, in Australian registries on the blind population, it accounts for 2% of blindness, highlighting the burden of this condition [6].

We report a case of LHON highlighting its peculiar, yet unresolved, feature of male predominance without any identified environmental triggers precipitating vision loss.

## Case presentation

A 15-year-old Sri Lankan male, a product of a nonconsanguineous marriage, presented with gradual-onset bilateral progressive painless vision loss that developed over 3 months. Initially, he had noted a dark spot in the center of his right visual field with color desaturation in the periphery that gradually progressed. The left eye was affected within 3 months of the onset of the right eye visual loss. There was no ocular pain on eye movement, headache, or other neurological symptoms. Detailed historical inquiry did not suggest any nutritional deficiencies, environmental toxins, or drug exposures, including smoking, alcohol, or other recreational drug abuse. His family history and past medical history were unremarkable.

Examination revealed that he only had perception to light in both eyes, associated with optic atrophy on funduscopy. The rest of the neurological and systemic examinations were normal. His biochemical and hematological investigations, and inflammatory markers were normal. Magnetic resonance imaging (MRI) of the brain and the orbits was normal. Cerebrospinal fluid (CSF) analysis was normal, while his serum did not have detectable anti-MOG or anti-AQP4 antibodies, which was analyzed to rule out the possibility of optic nerve demyelinating disorders.

**Fig. 1:**
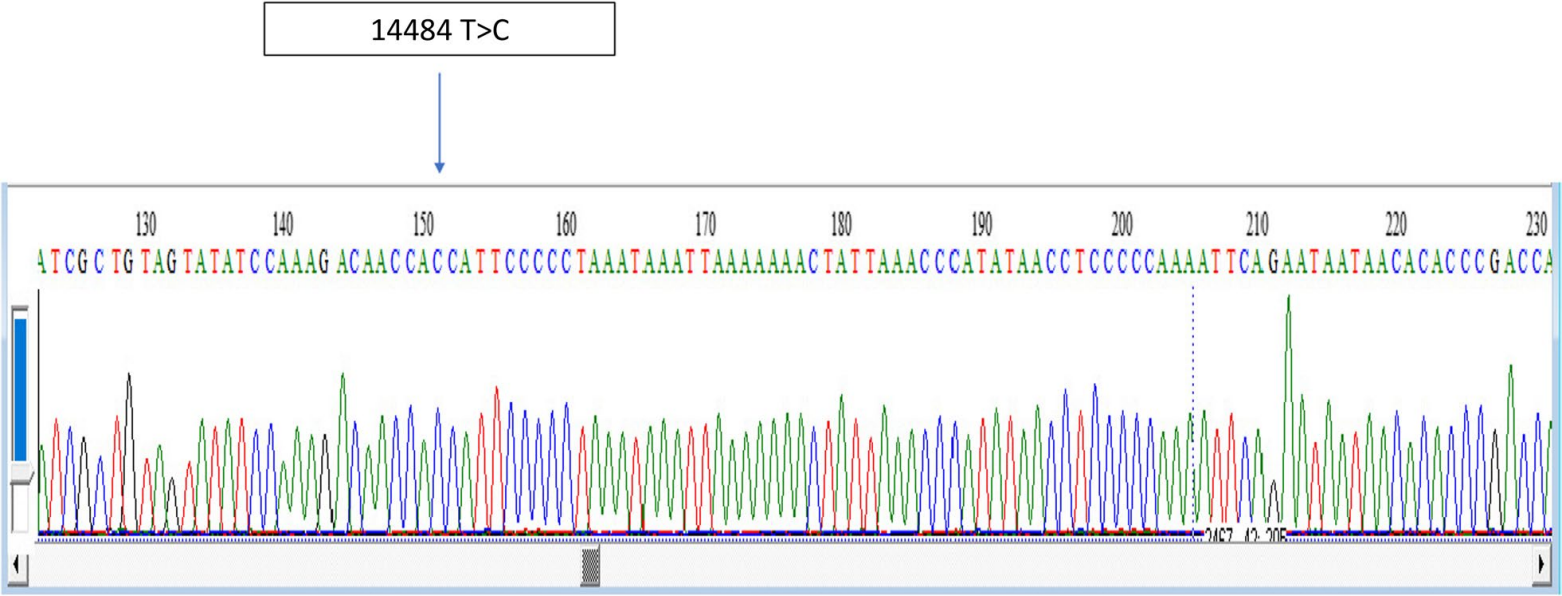
Electrophorogram of the patient produced via Sanger sequencing for the three common genetic variants depicting m.14484T > C variant in *MT-ND6* gene

**Fig. 2:**
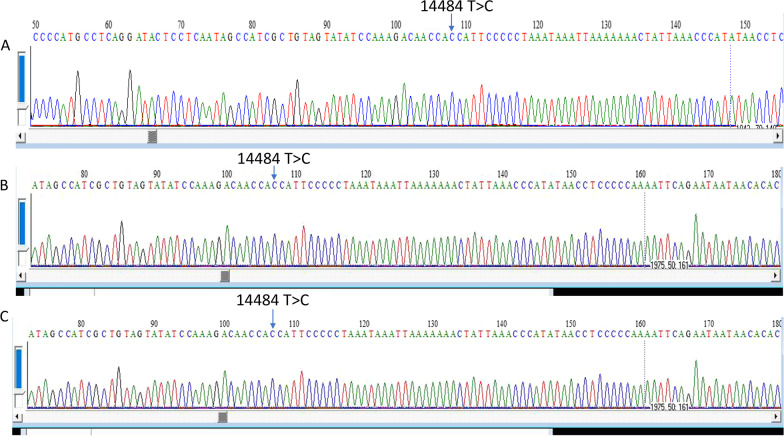
Electrophorograms produced by Sanger sequencing of the patient’s asymptomatic mother (**A**), elder female sibling (**B**), and younger female sibling (**C**) depicting the same genetic variant m. 14484T > C in *MT-ND6* gene with homoplasmy

Optical coherence tomography (OCT), done at 5 months of vision loss development, showed evidence of bilateral severe ganglion cell loss with macular thinning. Targeted mutational analysis for the three common mutations described in LHON revealed that the patient had a m.14484T > C mutation in *MT-ND6* gene, for which he was homoplasmic (Fig. 1), while whole exome sequencing did not reveal any other pathogenic variants implicated in visual impairment. Using Sanger sequencing, it was found that his mother (44 years of age), and two female siblings (22 years and 5 years of age), carried the same pathogenic variant and were all homoplasmic, despite not having any visual or funduscopic abnormalities (Fig. 2).

Currently, there is no curative or preventive treatment; the patient was offered only visual rehabilitation therapy, while post-test counseling was provided to the family. At review 1 year after the onset of his illness, no improvement was noted in his visual impairment.

## Discussion

LHON is a genetic disease of mitochondrial inheritance that is characterized by gradual-onset painless progressive bilateral vision loss. Development of visual impairment usually occurs during the second to third decades of life and rarely after 50 years of age [7]. However, cases have been reported within the age range of 2–87 years [8].

Bilateral visual impairment is seen in the majority with simultaneous (25%), or sequential (75%), involvement within a median duration of 8 weeks [9]. The early stage is characterized by impaired color perception with preserved pupillary reflex and fundoscopic evidence of vascular tortuosity of the central retinal vessels, circumpapillary telangiectatic microangiopathy, and swelling of the retinal nerve fiber layer [7]. Usually, the maximum visual deficit is reached between 4 and 6 weeks [7]. Optic atrophy is seen in almost all cases by 6 months [9].

Unaffected LHON pathogenic variant carriers manifest subclinical signs and symptoms, such as peripapillary microangiopathy, zones of mild disc pseudoedema, and telangiectasia on fundoscopy with subclinical dyschromatopsia [10].

Ninety-five percent of cases are accounted for by three pathogenic variants in the mitochondrial genome (Table 1) [11–13]. These variants affect the mitochondrial respiratory cycle, leading to selective retinal ganglion cell dysfunction and loss as they are highly sensitive to mitochondrial dysfunction and metabolic stress [7].

**Table 1:** Three common pathogenic variants in the mitochondrial genome implicated in Leber hereditary optic neuropathy and their prevalence (%)

Variant	Gene	Prevalence (%)
m.11778G > A	*MT-ND4*	69% (11)
m.14484T > C	*MT-ND6*	14% (12)
m.3460G > A	*MT-ND1*	13% (13)

Table 1 presents the three common mitochondrial gene variants implicated in LHON, their genes, and prevalence.

Genetic confirmation of the condition not only has a diagnostic value, but it also predicts visual recovery. Variants m.3460G > A in *MT-ND1* gene and m.11778G > A in *MT-ND4* gene are associated with significant impairment in visual function and poor visual recovery, whereas variant m.14484T > C in *MT-ND6* gene is associated with the best long-term visual outcome [14].

However, the visual recovery in our patient was poor. This may have been because we reevaluated the patient at 1 year, whereas in most patients with the variant m.14484T > C in *MT-ND6*, the visual improvement occurs later [15].

LHON has two interesting phenomena of incomplete penetrance and male predominance, which were both found in our patient [7]. This would explain a negative family history in about 40% of cases [16].

Heteroplasmy (having a mixture of wild-type and mutant alleles in the same individual) is one hypothesis being considered for incomplete penetrance with limited evidence [17]. In LHON, 10–15% of mutation carriers seem to be heteroplasmic [18]. In our case, all the asymptomatic female family members were homoplasmic, suggesting that the mutational burden may not be the sole factor deciding disease expression.

The existence of an X-linked susceptibility gene interacting in synergy with the mitochondrial pathogenic variant to cause male predominance of LHON has been studied extensively with some evidence, although not conclusive [19, 20].

Currently, there is some hope for visual recovery with the approval of idebenone, a synthetic short-chain benzoquinone, in some countries for the treatment of LHON [21]. This medication has demonstrated a trend towards improved visual acuity in a double-blind placebo-control clinical trial [21]. Gene therapy is also emerging as a treatment option for LHON, but evidence is still insufficient for its use in clinical practice [22].

## Conclusions

LHON is a mitochondrial disorder leading to blindness. Our case report is intended to increase awareness to consider it as a possible differential in a male patient presenting with sequential or simultaneous bilateral visual loss, even in the absence of a family history. Furthermore, our case suggests the possibility of an X-linked gene governing LHON disease expression, which warrants further investigation.

## Data Availability

The data generated and analyzed are not publicly available, but are available from the corresponding author on reasonable request.
